# Immunosenescence in neurocritical care

**DOI:** 10.1186/s40560-018-0333-5

**Published:** 2018-10-12

**Authors:** Shigeaki Inoue, Masafumi Saito, Joji Kotani

**Affiliations:** Department of Disaster and Emergency Medicine, Kobe University Graduate School of Medicine, Kusunoki-cho 7-5-2, Chuo-ward, Kobe, 650-0017 Japan

**Keywords:** Sepsis, Elderly, Immunosenescence, Immune paralysis

## Abstract

**Background:**

Several advanced and developing countries are now entering a superaged society, in which the percentage of elderly people exceeds 20% of the total population. In such an aging society, the number of age-related diseases such as malignant tumors, diabetes, and severe infections including sepsis is increasing, and patients with such disorders often find themselves in the ICU.

**Main body:**

Age-related diseases are closely related to age-induced immune dysfunction, by which reductions in the efficiency and specificity of the immune system are collectively termed “immunosenescence.” The most noticeable is a decline in the antigen-specific acquired immune response. The exhaustion of T cells in elderly sepsis is related to an increase in nosocomial infections after septicemia, and even death over subacute periods. Another characteristic is that senescent cells that accumulate in body tissues over time cause chronic inflammation through the secretion of proinflammatory cytokines, termed senescence-associated secretory phenotype. Chronic inflammation associated with aging has been called “inflammaging,” and similar age-related diseases are becoming an urgent social problem.

**Conclusion:**

In neuro ICUs, several neuro-related diseases including stroke and sepsis-associated encephalopathy are related to immunosenescence and neuroinflammation in the elderly. Several advanced countries with superaged societies face the new challenge of improving the long-term prognosis of neurocritical patients.

## Background

Japan is facing the social problem of a declining birth rate and an aging population, in which it is estimated that people aged at least 65 will constitute 30% of the total population by 2030. The average age of citizens is rising not only in Japan but also in advanced regions such as Europe and the USA, as well as in many Asian countries such as China and South Korea. It is predicted that by 2050, most of the world’s population except for Africa and the Middle East will be at least 65 years old. With the percentage of elderly people exceeding 20%, we are entering a superaged society [1]. In such an aging society, various diseases such as malignant tumors, diabetes, and severe infections are increasing, and patients with such disorders often find themselves in an intensive care unit (ICU). These diseases are closely associated with age-related immune dysfunction so-called immunosenescence.

## What is the immune system?

Immunity is the means by which multicellular organisms resist the attacks of harmful invading microorganisms. Such immunity is achieved by two systems: innate immunity and adaptive immunity.

The innate immune system mainly comprises innate immune cells (macrophages: neutrophils, dendritic cells) and complement factors. Innate immune cells are also called phagocytes because they phagocytose when they recognize foreign substances such as lipopolysaccharides (LPSs). Complement factors circulate in the blood and are activated by the membrane of the microorganism to directly destroy the pathogen or activate phagocytic cells indirectly to eliminate the pathogen. The innate immune system is activated within several hours of encountering pathogens, etc. However, the efficiency of this activation is not affected by previous infections.

In contrast, the adaptive immune system consists primarily of T and B cells and in theory can eliminate an infinite variety of targets. Although the acquired immune system functions as early as 2–4 days after encountering the pathogen, some T and B cells respond specifically to the invading microorganisms, even after the immune response has ended. The response is maintained as an immune memory and can be activated quickly when subsequent encounters with the same pathogen occur.

Because CD4 + T cells, which constitute the “control tower” of acquired immunity, cannot recognize microbial components such as LPSs, the acquired immunity response to microorganisms depends on the phagocytic cells of the innate immunity. This role is fulfilled by specialized cells called dendritic cells. When dendritic cells are activated by inflammatory cytokines such as LPSs and inflammatory cytokines produced by macrophages, they present fragments of pathogens digested intracellularly to T cells and induce the activation of antigen-specific T cells. During that process, naive T cells are stimulated and differentiated into effector T cells that can kill cells or activate other cells. Effector T cells activate B cells, so that B cells produce antibodies that recognize microorganisms.

## Immunosenescence

Aging is a biological change that occurs in individuals over time and involves a decline in function and processes that is particularly apparent as the organism dies. This is a biological process that is common to all living things. Our bodies undergo functional deterioration with organic changes at various sites depending on aging. There are various theories about the aging mechanism, but telomere shortening always accompanies aging. Oxidative stress induced by molecular species such as active oxygen damages the genome, and somatic cells are thought to cause senescence-related protein accumulation and senescence. The immune system is similarly affected, and the immune response in normal individuals is dependent on aging. Because the prevalence of malignant tumors and infectious diseases increases with an age-related decline in immune function, it is presumed that there is some relationship between this reduction in immune function and the onset of these diseases.

**Fig. 1:**
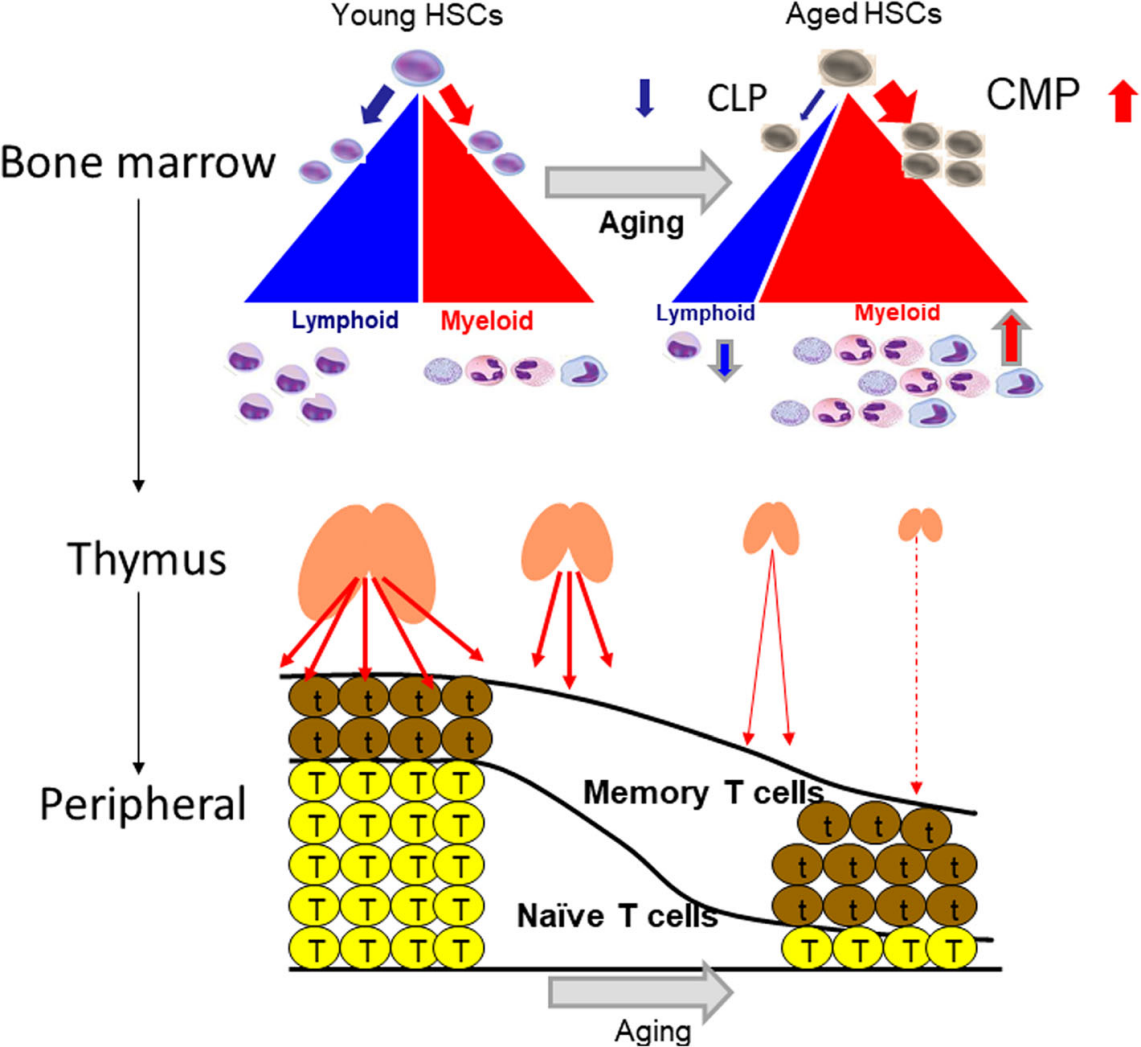
Changes in bone marrow/thymus accompanying aging and changes in immune response cells. Although the number of stem cells in the bone marrow is not affected by aging, differentiation into common lymphoid progenitor cells decreases and shifts to differentiation into myeloid-type common progenitor cells. Therefore, differentiation into lymphoid cells (T cells, B cells) decreases, and differentiation into myeloid cells (granulocytes/monocytes) increases. The thymus, which is the site of the differentiation and maturation of T cells, atrophies with age. Therefore, in young people, naive T cells predominate; however, with age, there is a shift to dominant T cells (memory T cells), which is activated by antigen stimulation or some internal factor. HSCs, hematopoietic stem cells; CMP, common myeloid progenitor; CLP, common lymphoid progenitor

The efficiency and specificity of the immune system decline with age. The most noticeable change in immune function associated with aging is a decrease in antigen-specific acquired immunity. Although elderly people generally retain pathogen-specific immune memory obtained when young, the efficiency of their response to new infections and vaccines is often low. Another characteristic is that senescent cells accumulate in body tissues over time and cause chronic inflammation. This is known as the senescence-associated secretory phenotype (SASP) and is described later [2]. The chronic inflammation accompanying such aging is called “inflammaging” (inflammation + aging), and its relationship with age-related disease is attracting increasing attention [3, 4]. The functional changes to the immune system that accompany aging are generally called immunosenescence. Hematopoietic stem cells are the source of all immune response cells, but their numbers in the bone marrow are not affected by aging. However, the differentiation of hematopoietic stem cells into lymphoid common precursor cells decreases and shifts toward differentiation into myeloid-type common progenitor cells over time [5, 6]. Therefore, differentiation into lymphoid cells (T cells, B cells) decreases and differentiation into myeloid cells (granulocytes/monocytes) increases (Fig. 1). The roles of each immunocompetent cell and the changes associated with aging are described below.

## Innate immunity (Fig. 2)

### Neutrophils

**Fig. 2:**
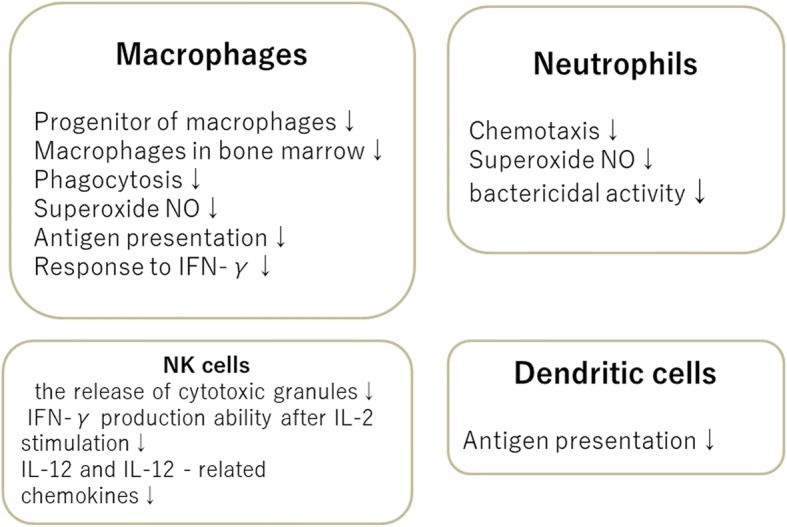
Age-related changes in innate immune effector cells

Neutrophil is an essential part of the innate immune, which is chemotactic with regard to cytokines and pathogens such as bacteria and fungi. They infiltrate the inflamed region to engulf, disinfect, and decompose foreign substances including bacteria and fungi and are the main protagonist of inflammation and immunity in the early stages of infection. Neutrophils experience less pronounced changes than T cells with age, and there is no change in the expression level of receptors that are important for intracellular signal transduction factors such as neutrophil count, phagocytosis capability, and toll-like receptors 2 and 4. However, aging is accompanied by reduced superoxide and chemotaxin production and by a decline in bactericidal activity [7] (Fig. 2).

### Macrophages

Macrophages are chemotactic phagocytes that move around the body like amoeba. They decompose and digest foreign bodies such as dead cells and their fragments, and invading bacteria. Macrophages have antigen-presenting capability and activate CD4 + T cells by fragmenting degraded foreign matter and presenting it to them. As with neutrophils, the number of macrophages is not affected by aging, but phagocytic activity, and the production of superoxide and nitric oxide (NO) do decline with age [8, 9]. Moreover, activation is impaired in the macrophages of elderly mice, i.e., the ability to present antigens to T cells is reduced [9, 10] and reactivity with interferon-γ (IFN-γ) declines [10].

### Dendritic cells

Dendritic cells, which is a generic term for unspecified cells that exhibit dendritic morphology, have become widely known as antigen-presenting cells in recent years. They are present in tissues that come into contact with the exterior environment, including the skin, the nasal cavity, the lungs, the stomach, and the intestinal tract. They process antigens from microorganisms and promptly present them to CD4 + T cells, thereby acting as a link to acquired immunity. Recently, the observation that dendritic cells change with age has led to the suggestion that the number of Langerhans cells decreases in the elderly. The migration of dendritic cells to lymph nodes is impaired in elderly mice [11], and it has been reported that major histocompatibility complex 2(MHC2), CD80/86, and other molecules are expressed less and have impaired antigen-presenting capability [12].

### Natural killer (NK) cells

NK cells are cytotoxic lymphocytes that make an indispensable contribution to innate immunity. They are particularly important for eliminating tumors and virus-infected cells. Although it is not clear how the reduction in the number of NK cells is linked to aging, the release of cytotoxic granules and the decrease in IFN-γ production capability after stimulation with interleukin-2 (IL-2), IL-12, and IL-12-related chemokines [macrophage inflammatory proteins-1a (MIP-1a), regulated on activation, normal T cell expressed and secreted (RANTES), IL-8] reduce the production of NK cells [13]. Therefore, it is possible that virus removal in the early stages of infection may be impaired by aging [14]. A reduction in NK activity associated with aging has been reported in patients with oral candidiasis, and it has been suggested that aging is involved in the onset and progress of the disorder [15].

## Adaptive immunity (Fig. 3)

### B cells

**Fig. 3:**
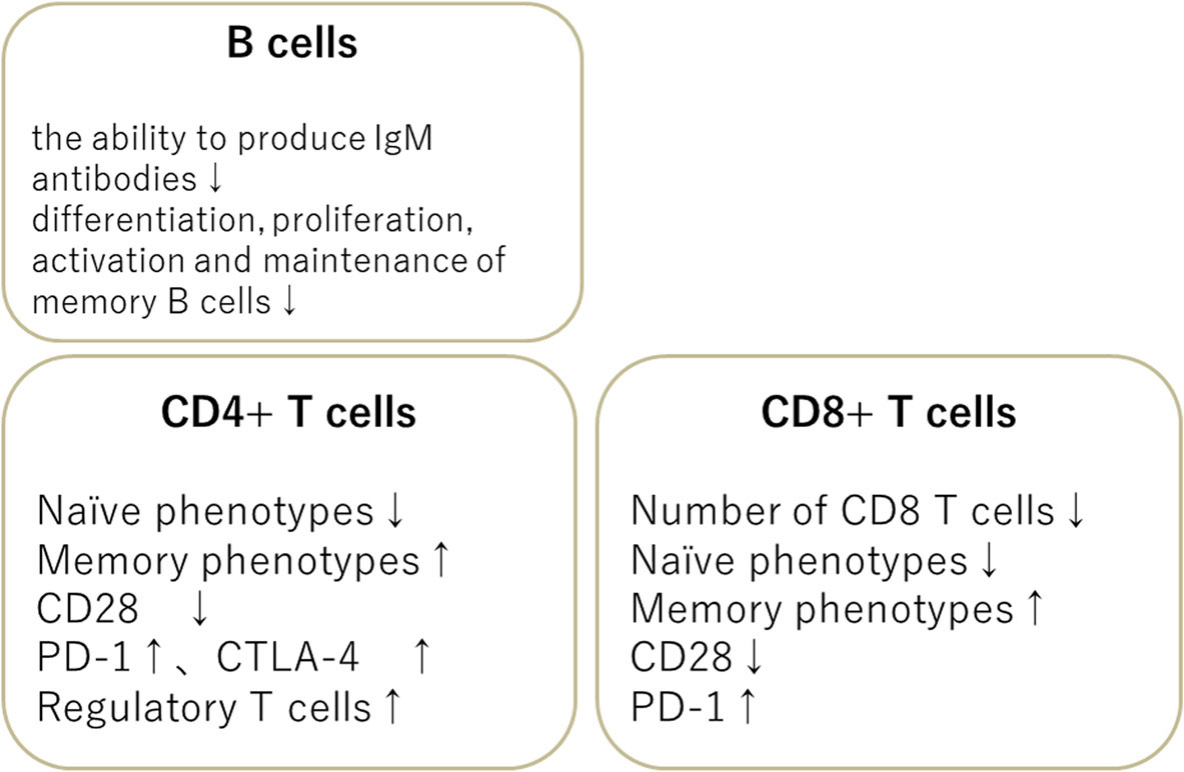
Age-related changes in adaptive immune effector cells

B cells proliferate in response to antigen invasion and differentiate into plasma cells that produce antibodies (immunoglobulins). They are also affected by aging. For example, in the elderly, the ability to produce immunoglobulin M (IgM) antibodies decreases, and IgM antibody titers are also lower than that in healthy adults after ingesting pneumococcal vaccine [16, 17]. Antibody production capacity for influenza vaccines is also approximately 50% of that in healthy adults [18, 19]. The reason for this is that differentiation, proliferation, activation, and maintenance of memory B cells are impaired in the elderly [20] and, as described above, functional disorders of CD4 + T cells associated with aging affect B cell activation [21] (Fig. 3).

### T cells

The most dynamically age-dependent change with regard to immunity occurs in the thymus, which plays an important role in the differentiation and maturation of T cells. In humans, thymic epithelial tissues gradually become atrophied during adolescence, are replaced with adipose tissue, and become almost fatty remnants from maturity to old age. The thymus is a primary lymphoid organ that serves as a site of differentiation, maturation, and the selection of T cells from inflowing hematopoietic stem cells, suggesting that the generation of new functional mature T cells and their supply to the periphery is affected by age. This means that the activity of the thymus rapidly declines (Fig. 3). There is a greater proportion of naive T cells that have not yet received antigen stimulation in the young compared with T cells activated by antigen stimulation or some internal factor (memory T cells), which are predominant in the old. Furthermore, the length and activity of the telomeres within T cells, the responsiveness to cytokines that activate T cells such as IFN-γ and interleukin-2 (IL-2), and decreased proliferation of T cells are associated with aging [22, 23].

T cells are roughly divided into CD4 + T cells and CD8 + T cells. CD4 + T cells are activated by antigen presentation from macrophages, dendritic cells, etc., and act as controllers of the acquired immune system. During its activation, CD28—a surface antigen of T cells—plays an important role as a costimulatory molecule. CD4 + T cells are activated via CD28 to become effector T cells, but the prevalence of CD28 on T cells decreases with age [24], T cell activation disorder, viruses, etc. [25]. In contrast to the effects of CD28, T cell activity is suppressed via surface receptors such as programmed cell death protein 1 (PD-1) and cytotoxic T-lymphocyte-associated protein 4 (CTLA-4).

#### T cell exhaustion in elderly patients with sepsis

Although the mechanism by which immunosuppression takes place after septicemia remains unclear, Hotchkiss et al. confirmed that the number of lymphocytes decreases owing to apoptosis in sepsis patients [26]. In addition to the lymphocyte count, attention has recently been focused on T cell dysfunction after sepsis, i.e., T cell exhaustion. T cell exhaustion means narrowing of the T cell antigen receptor (TCR) repertoire due to long-term exposure to antigens, decreased TCR signaling, and reduced levels of PD-1 and CTLA-4. The T cells are in a dysfunctional state as a result of the induction of various co-suppressive molecules, such as CTLA-4 and T cell immunoglobulin and mucin-domain containing-3 (TIM-3), and disorders in IL-2 production, activation, and proliferation [27–29].

In a previous study conducted by this research team, we found an increase in the level of PD-1-positive T cells and reduced IL-2 production, activation, and proliferation in elderly sepsis patients and older mouse sepsis models [30]. In the acute phase within 0–2 days after septicemia diagnosis, the rate of bacterial infection of the blood was similar in elderly and young patients, but 2 and 4 weeks after septicemia the rate of bacterial infection was higher in the elderly than in the young. In comparison, the opportunistic infection by attenuated pathogens such as *Acinetobacter* species, *Stenotrophomonas maltophilia*, and *Candida albicans* increased. Based on the above, we think that T cell exhaustion and death during subacute periods in elderly patients with sepsis are related to an increase in nosocomial infections after septicemia.

#### Aging and chronic inflammation

**Fig. 4:**
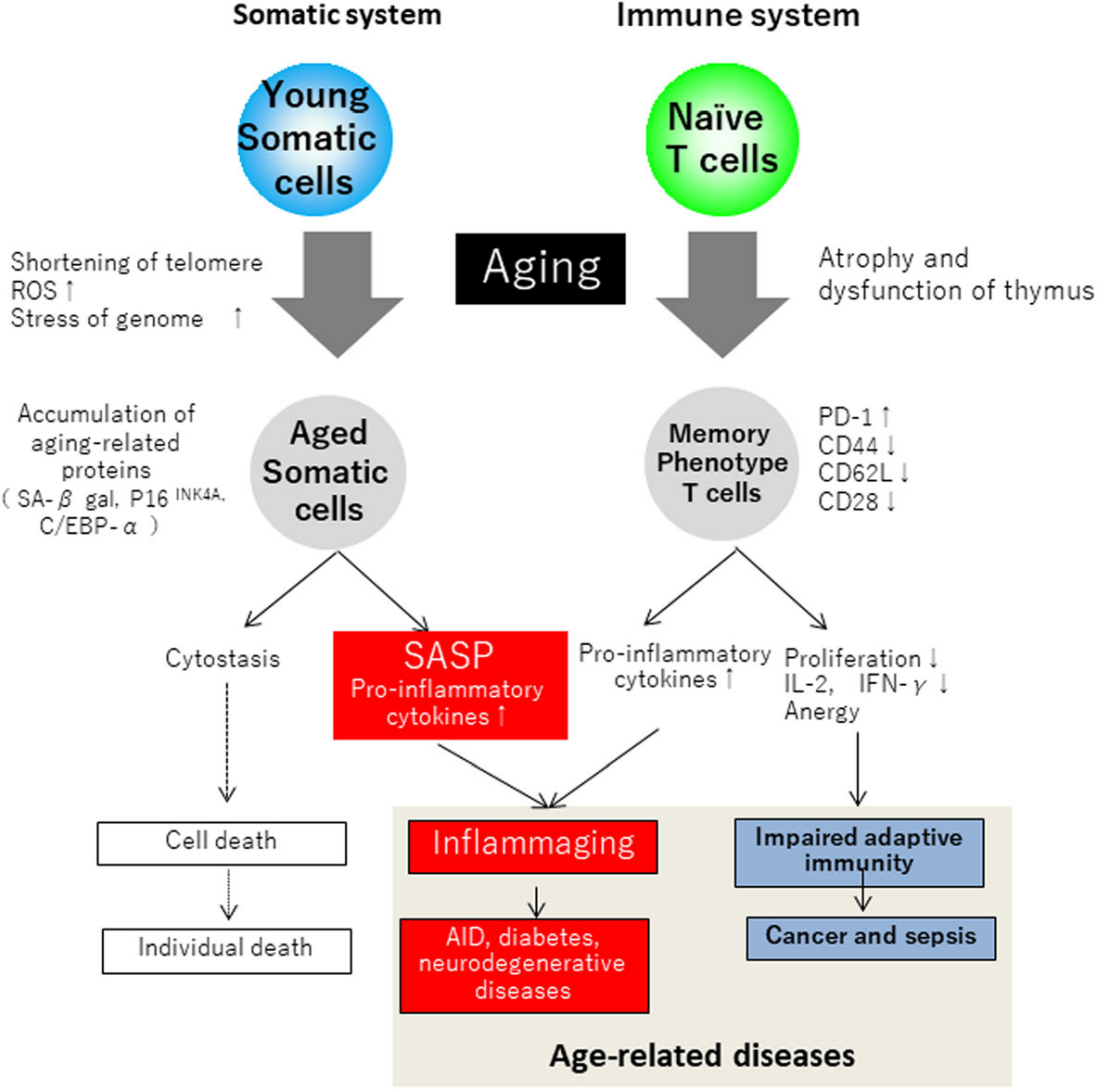
Aging of somatic cells and immune effector cells. SAPS, senescence-associated secretory phenotype

The SASP hypothesis, in which the senescent cells that accumulate in body tissues over time contribute to inflammation progression in the elderly, has recently been proposed [2]. First, during aging, the p53/RAS/pl6 signaling pathway is activated by DNA damage, reactive oxygen species (ROS) accumulation, telomere shortening, and cellular senescence. This produces the SASP phenotype, which secretes inflammatory cytokines such as IL-1β, IL-6, and IL-8, and vascular growth factors such as vascular endothelial growth factor. Further cell senescence and chronic inflammation of the surrounding cells are thought to be prolonged by this phenotype [2]. Persistent chronic inflammation that is not related to such infection is a fundamental pathology of various diseases such as obesity, diabetes, cancer, neurodegenerative diseases, and autoimmune disorders. The incidence of diseases associated with various chronic inflammatory pathologies increases with age (Fig. 4).

Epidemiological studies of the elderly beginning in the 1990s have revealed that the prevalence of inflammatory markers such as C-reactive protein (CRP) increases with age and correlates with mortality and inflammaging. The relationship between age and disease has been noted. By specifically eliminating cells with p16^INK4A^, which is a biomarker of aging, the development of age-related diseases can be delayed in the tissue (e.g., adipose and muscle tissue) of a senescence-promoting mouse model. Moreover, it is possible to directly cause aging of tissues and individuals [31, 32]. A long-term therapeutic strategy aimed at improving the quality of life of very old patients, which may involve molecular biology, will become increasingly important.

#### Aging in neurocritical care

Neurointensive care is an area of medicine that spans multiple fields and provides specialized care for critically ill patients with neurological illnesses [33, 34]. Neurointensivists are clinical professionals who orchestrate personnel including neurologists, neurosurgeons, consultants, therapists, pharmacists, nurses, and administrators in neurosciences intensive care units (NSICUs). Their role is important for the patient’s health and clinical outcomes [35, 36]. Studies have shown that neurointensivists who manage NSICUs improve outcomes and documentation and shorten the length of stay of all neurocritically ill patients [37–41], including those suffering from ischemic stroke [41–46], subarachnoid hemorrhage [47, 48], traumatic brain injury [49, 50], intracerebral hemorrhage [51], and neuromuscular respiratory failure [52].

#### Stroke and T cell dysfunction

Stroke remains a leading cause of death and disability worldwide and is a major problem in neurocritical care. Ischemic stroke is characterized by the disruption of cerebral blood flow, which produces a central core of dead neurons surrounded by a penumbra of damaged but partially functional neurons [53].

T lymphocytes are central to the development of a sustained inflammatory response, and there is evidence that they accumulate in the post-ischemic brain within a few hours of reperfusion [54, 55]. Profound systemic immunodepression—or “stroke-induced immunodeficiency syndrome”—occurs as early as 12 h after ischemic stroke and may persist for several weeks [56–60]. This phenomenon is characterized by reduced numbers of T cells and other immune cells of the spleen, thymus, and lymph nodes and is mediated by hyperactivity of the sympathetic nervous system (SNS) and the hypothalamic–pituitary–adrenal axis (HPA) [60]. This leads to increased apoptosis of immune cells in the spleen, thymus, and lymph nodes, and as a result, these secondary lymphatic organs undergo atrophy [59, 60]. Furthermore, there is a shift from the production of cytokine Th1 to that of Th2 [60, 61]. Infectious complications, predominantly chest and urinary tract infections, occur in many stroke patients within the first few days of the stroke, and the development of an infection soon after the stroke is associated with worse outcomes [62–64]. Several recent clinical studies have found evidence that SNS-mediated stroke-induced immunodepression and subsequent susceptibility to post-stroke infections also occur. In the PANTHERIS (Preventive Antibacterial Treatment in Acute Stroke) trial on the efficacy of short-term antibacterial therapy to prevent the development of post-stroke infections, Klehmet et al. confirmed that rapid loss and functional deactivation of T cells are common in stroke patients and are consistent with immunodepression following brain ischemia. Furthermore, a more pronounced decline in cellular immune responses and increased sympathetic activity following stroke are associated with a higher risk of infection [65]. Harms et al. conducted a post hoc analysis of the PANTHERIS trial by investigating the impact of distinct lesion patterns on SNS activation, immunodepression, and frequency of post-stroke infections [66]. Large stroke volume, lesions affecting distinct regions of the middle cerebral artery (MCA) cortex, and SNS activation (assessed by elevated norepinephrine levels) were all associated with impaired immune function and higher susceptibility to post-stroke infections. Whereas neither stroke severity nor stroke volume was independently associated with post-stroke infections, increased levels of norepinephrine and infarction of the anterior MCA cortex were both identified as independent risk factors for post-stroke infections [66]. A recent study by Hug et al. [67] found that reduced costimulatory efficacy of circulating costimulatory cells (i.e., splenic non-T cells) in mice is an important feature of stroke-induced immunodepression and, if confirmed in humans, points to such cells as potential targets for therapies to prevent secondary inflammatory damage to the brain after stroke. In addition to the well-established proinflammatory cytokine-mediated activation of the SNS and HPA, another pathway of communication between the nervous and immune systems, known as the vagal cholinergic anti-inflammatory pathway, has been identified. When the vagus nerve is activated by proinflammatory cytokines, it releases acetylcholine, which inhibits the release of more proinflammatory mediators by macrophages [68–70]. Experimental studies have shown that, according to various models of ischemia-reperfusion, vagal nerve signaling inhibits the release of proinflammatory cytokines and improves outcomes [70]. Taken together, the vagal cholinergic anti-inflammatory pathway is another potential mediator and therapeutic target of stroke-induced immunodepression.

#### Sepsis-associated encephalopathy (SAE)

Sepsis is one of the most common reasons for presentation to emergency departments and accounts for 6.4% of admissions [71, 72]. Sepsis and its attendant complications cause more deaths than prostate cancer, breast cancer, and HIV/AIDS combined and impose a major financial burden on healthcare systems.

Age increases the risk of mortality in sepsis patients [73]. Elderly people aged at least 65 account for approximately 60% of sepsis patients and approximately 80% of deaths due to sepsis [74]. The average age of sepsis patients in many developed countries is rising each year. In recent years, diseases closely related to physical dysfunction of the elderly, such as ICU-acquired weakness and post-intensive care syndrome, have also been proposed, and the subjects of intensive care in the twenty-first century are aging.

SAE is a multifactorial syndrome that is characterized by diffuse cerebral dysfunction induced by the systemic response to infection without clinical or laboratory evidence of direct brain infection or other types of encephalopathy (e.g., hepatic or renal encephalopathy). Brain dysfunction due to sepsis has been overlooked as a cause of delirium or altered mental status in critically ill patients. This is primarily because there are no precise, well-established clinical or biological markers of damage to assess brain dysfunction occurring because of sepsis [75]. However, the authors of recent studies have reported that SAE is a relatively common cause of altered mental status in critically ill patients admitted to ICUs, and its prevalence varies from 8 to 70% [76–78]. The clinical spectrum of SAE may range from mild inattentiveness or disorientation, agitation, and hypersomnolence to more severe disruption of consciousness, as seen in coma. Although there is no direct infection or invasion of the central nervous system (CNS), laboratory evidence of CNS dysfunction is common in SAE. The pathophysiology of SAE has not been established, but several likely mechanisms have been proposed [79]. SAE appears to involve direct cellular damage to the brain, mitochondrial and endothelial dysfunction, neurotransmission disturbances, and derangements of calcium homeostasis in brain tissue [80]. The direct local cerebral colonization of microorganisms and the formation of micro abscesses have been described in human SAE [78]. However, many cases of SAE without brain micro abscesses have been observed; there is no correlation between SAE and any microorganism, making it unlikely that microorganisms play a causative role in SAE.

#### Breakdown of blood–brain barrier (BBB) in SAE

**Fig. 5:**
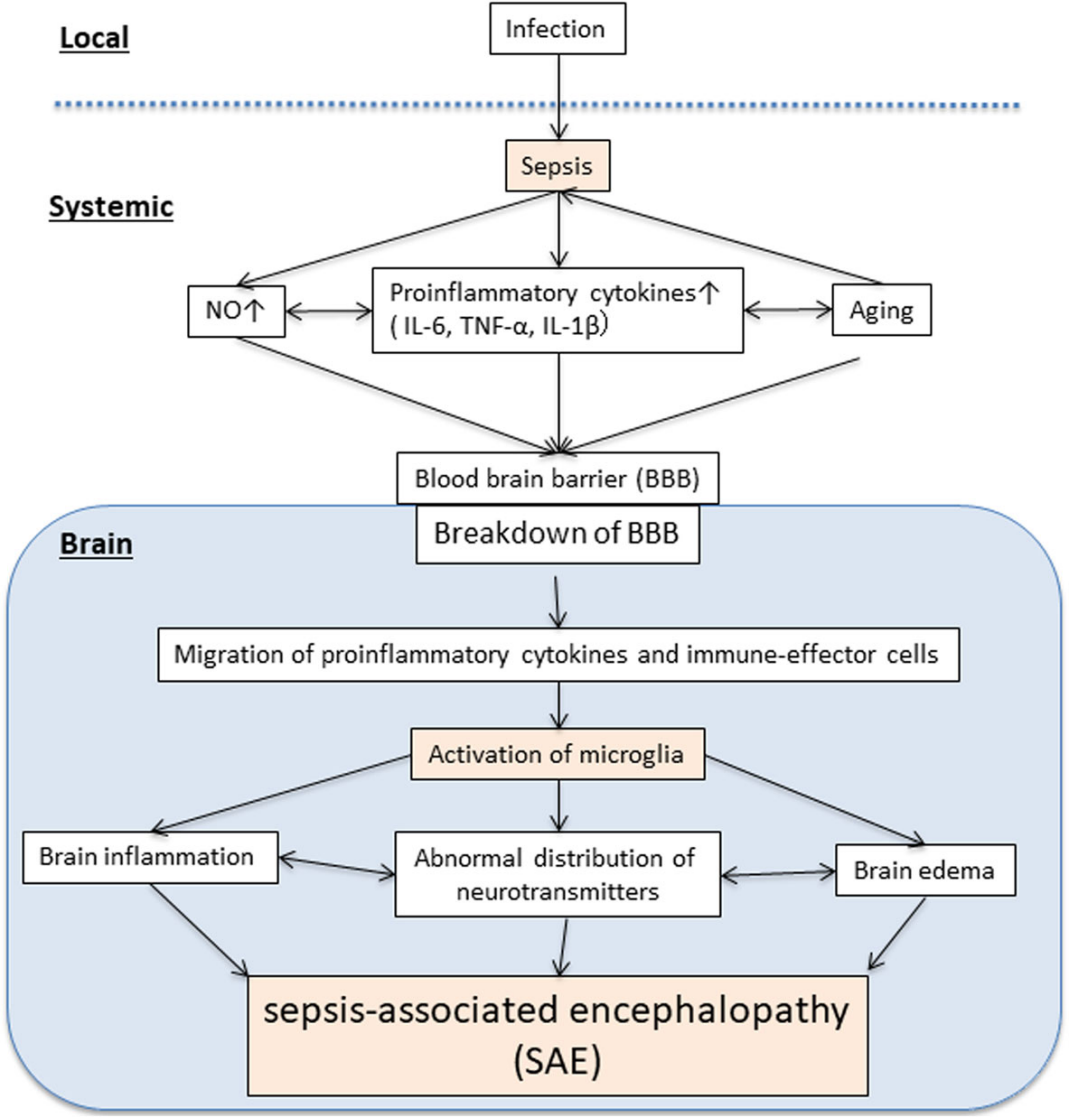
Mechanism of sepsis-associated encephalopathy

Adequate function of the cerebral microcirculation and BBB is important for the maintenance of normal cerebral function. The BBB, which comprises endothelial cells, astrocytes, and pericytes, plays a central role in maintaining the vascular homeostasis of the CNS [81]. Experimental data indicate that, in the early phase of sepsis, endothelial nitric oxide synthase-derived NO exerts proinflammatory effects and contributes to the activation and dysfunction of cerebrovascular endothelial cells [82]. Secondly, LPSs and cytokines induce the expression of adhesion molecules on brain microvessel endothelial cells, which also contributes to BBB dysfunction. This breakdown of the BBB facilitates the passage of neurotoxic factors such as cytokines and accounts for the brain edema revealed by magnetic resonance imaging (MRI) in patients with SAE [83]. Inflammatory cytokines and the complement system constitute the final common pathway in the pathophysiology of brain dysfunction in SAE (Fig. 5). TNF-α appears to be one of the most significant inflammatory mediators in SAE. It induces neutrophil infiltration of the brain tissue, neuronal cell apoptosis, and brain edema (probably by inducing the expression of aquaporin-4) [84]. IL-6 also plays a crucial role in the pathogenesis of SAE. Excessive complement activation can cause altered expression of TLR4 and subsequent alterations in TNF-α, inducible nitric oxide synthetase (iNOS), and aquaporin-4, thereby causing edema, cell necrosis, or neuronal apoptosis [80, 85].

#### Aging induces breakdown of the BBB

In the aging population, common cardiovascular disorders such as hypertension [86], seizure [87], and stroke [88] contribute to BBB dysfunction. BBB permeability is altered by several factors including increased levels of inflammatory cytokines [89] and free radicals [90], which cause the increased influx of cytokines and immune cells into the brain. Moreover, dysfunction of the endothelial barrier facilitates extravasation of plasma proteins into the brain and subsequently triggers a variety of neuroinflammatory responses within the brain. Aging is associated with degeneration of the BBB/blood cerebrospinal fluid barrier, and the abnormal accumulation of albumin [91], fibrinogen, and IgG has been reported in the brains of patients with Alzheimer’s disease [92]. Taken together, these observations suggest that aging induces the progression of SAE via BBB dysfunction in elderly patients with sepsis.

## Conclusions

Advances in medical science, especially developments in intensive care medicine, have increased the lifespan of human beings, and aging has become a global issue. Several diseases, including stroke and sepsis-induced encephalopathy, are closely related to aging-induced immune dysfunction, and the terms “immunosenescence” and “inflammaging” are sometimes used in neurocritical care units. Several advanced countries, which now have superaged societies, face the new problem of improving the long-term prognosis of neurocritical patients.

## Abbreviations

BBB: Blood–brain barrier
CNS: Central nervous system
CRP: C-reactive protein
CTLA-4: Cytotoxic T-lymphocyte-associated protein 4
HPA: Hypothalamic–pituitary–adrenal axis
ICU: Intensive care unit
IFN-γ: Interferon-γ
IgM: Immunoglobulin M
IL-2: Interleukin-2
iNOS: Inducible nitric oxide synthetase
LPSs: Lipopolysaccharides
MCA: Middle cerebral artery
MHC2: Major histocompatibility complex 2
MIP-1a: Macrophage inflammatory proteins-1a
MRI: Magnetic resonance imaging
NSICUs: Neurosciences intensive care units
PD-1: Programmed cell death protein 1
RANTES: Regulated on activation, normal T cell expressed and secreted
ROS: Reactive oxygen species
SAE: Stroke and sepsis-associated encephalopathy
SASP: Senescence-associated secretory phenotype
SNS: Sympathetic nervous system
TCR: T cell antigen receptor
TIM-3: T cell immunoglobulin and mucin-domain containing-3
